# Finding links between organisation’s culture and innovation. The impact of organisational culture on university innovativeness

**DOI:** 10.1371/journal.pone.0257962

**Published:** 2021-10-08

**Authors:** Julia Gorzelany, Magdalena Gorzelany–Dziadkowiec, Lidia Luty, Krzysztof Firlej, Martina Gaisch, Oksana Dudziak, Cornelia Scott

**Affiliations:** 1 Department of Land Management and Landscape Architecture, University of Agriculture in Krakow, Krakow, Poland; 2 Department of Organization Development, Cracow University of Economics, Krakow, Poland; 3 Department of Statistics and Social Policy, University of Agriculture in Krakow, Krakow, Poland; 4 Department of Higher Education Research & Diversity Management, School of Informatics, Communications and Media, University of Applied Sciences, Hagenberg, Austria; 5 Department of Management, Administration and Public Management, State Agrarian and Engineering University in Podilya, Kamianets-Podilskyi, Ukraine; 6 Department of Economics and Business, Anhalt Uni of Applied Sciences, Bernburg, Germany; University of Salento, ITALY

## Abstract

The objective of the paper is to diagnose organisational culture of selected universities and analyse its impact on the innovation processes within them. The subject matter of the study was organisational culture and innovation at universities. The subjects were four selected universities in Poland, Austria, Germany, and Ukraine. The paper provided a definition of organisational culture and its typology. It further discussed the organisational culture of universities and the relationships between organisational culture and innovativeness. The literature review provided foundations for building a model for the formation of a type of organisational culture at universities that is innovation-friendly, which is the added value of the paper. It offers actions worth taking to shape innovation-friendly culture at universities. It is particularly important during difficult time of changing labour market, when universities greatly impact the attitudes of young people. The knowledge of how to shape innovation-friendly organisational culture at universities is necessary for academia to profile future employees in times of continuous changes. To investigate the relationship between organisational culture and the innovativeness of universities, we designed an original survey questionnaire [S1 File]. Organisational culture was diagnosed with the Organizational Culture Assessment Instrument by K.S. Cameron and R.E. Quinn. The analyses were conducted in Dell Statistica v. 13.1 (StatSoft Polska). We normalised data from the Likert rating scale using Kaufman’s and Rousseeuw’s formula. We used Spearman’s correlation coefficient and Kendall’s W to calculate correlations. The research shows that the investigated Polish and Austrian universities are dominated by hierarchy and market cultures. On the other hand, the German and Ukrainian universities host all cultures, but clan and adhocracy dominate there. Moreover, the analyses demonstrated that although the adhocracy culture was the least visible in the investigated organisations, it contributes to university innovativeness the most. The conclusions were used to build a model for promoting innovation-friendly organisational culture at universities. The model contains answers to the research questions. In addition, it offers guidelines for shaping organisational culture to bolster innovation at universities. The research identified relationships between organisational culture and university innovativeness and components that create innovation opportunities at universities as its contribution to management theory. When applied in practice, the guidelines can help form the university’s organisational culture bottom-up.

## Introduction

Culture is an important problem in management, which remains open and ambiguous both in theory and practice. The essence of the problem is related to its impact on the organisation’s functioning in its entirety. Culture is the environment where people and organisations act and the foundation for governance, valuation, and communication. Cultural processes within organisations have been investigated by management sciences for decades. On the one hand, it is related to the societal impact on work processes (stemming from social relationships), on the other hand, cultural differentiation of societies and its impact on management have been studied since the 1970s [1].

Organisational culture has been discussed rather broadly in the literature of the last decades of the 20^th^ century. Deshpande and Webster [2] described organisational culture as a model, shared values, and beliefs that help understand how an organisation functions and thus provide it with standards of conduct. Barney [3] and Slater and Olson, and Finnegan [4] explained that not only does organisational culture reflect who the employees, customers, suppliers, and competitors are, but it also defines how the company will work with key stakeholders. The right organisational culture helps employees understand the business strategy, motivates, and nourishes interpersonal relationships.

University organisational culture is a special case of organisational culture. It dates back to Antiquity (the Platonic Academy) and Medieval student and professor corporations. Historically, organisational culture was defined by such values and the master-pupil relationship, truth, knowledge, autonomy, independence, freedom, supernationality, and community. One could, therefore, argue that the emergence of the university as an organisation is a result of institutionalisation of a culture founded on traditional academic values and principles.

Moreover, organisational culture has become a means for pursuing university’s mission over centuries [5]. Literature offers publications on higher education in the context of organisational culture research. In 2015, Guthrie and Dumay [6] advocated continuous research on phenomena related to changes in social, political, and economic circumstances that would affect the future functioning of the public sector. The multitude of variables relevant to organisational culture that were investigated led to the notion of innovation-friendly culture. However, it is still to be included in the management theory [7]. When combined with the views of Fralinger and Olson [8] that organisational culture is the basic factor for decision-making at universities, the reflections above led to the determination of the background for the study and an inspiration for further investigations.

The university is one of the oldest and most complex organisations ever created by humans pursuing goals regarding research, education, and social impact that are often contradictory. The university of today is full of tension. It is suspended in a limbo between tradition and modernity. Some researchers believe that the mission of higher education as the institution for bringing up and nurturing the nation to strengthen the state is dwindling in the economic, cultural, and social environment that is growing ever more globalised [5]. Universities all over the world face numerous challenges, such as reduced public funds; changing requirements of stakeholders (such as students, business, government); the rapid growth of new technologies that change the foundations of education, research, and relationships with the surroundings (such as networking); the focus on effectiveness and efficiency (‘value for money’); the emphasis on perfection in education and research; and the urge for a greater social impact [1]. The factors listed above dramatically change the internal and external context of university management.

The foundations laid above led to identifying a research gap to find connections between organisational culture and innovativeness in higher education institutions. The paper’s objective is to diagnose organisational cultures at selected universities and analyse their impact on the innovation processes within them. The research question was how to form organisational culture that will promote innovation at universities. Additionally, we sought to answer the question what type of organisational culture dominates four selected Polish, Austrian, German, and Ukrainian universities and what is the relationship between a specific organisational culture type and the innovativeness of the universities. The subject matter of the study was organisational culture and innovation at universities. The subjects were four selected universities in Poland, Austria, Germany, and Ukraine.

The literature review provided foundations for building a model for the formation of a type of organisational culture at universities that is innovation-friendly. The matter is of interest both to researchers and practitioners of academia. The research looks into a far-reaching problem of promoting innovation in higher education through proper organisational culture. We attempted to investigate relationships between organisational culture and innovation and propose a way to form university organisational culture to foster its innovativeness.

The research was based on Polish and international literature, reports, and statistical compilations. The sources are complemented with original research using descriptive statistics and correlation analysis.

The ability to introduce innovation is a primary driver of survival and growth in the context of modern organisations. The labour market is evolving, and higher education has a great impact on the attitudes of young people. Therefore, knowledge of how to shape organisational culture is indispensable to design organisational changes. Innovation-friendly culture is transdisciplinary. It pragmatically integrates everything desirable, necessary, useful, feasible, and proper. Through appropriate organisational culture, one can stimulate innovation processes not only in business but also at universities. Therefore, organisational culture should be set to design a structure and behaviour that generate innovation. Regarding the value to the public, the knowledge presented in the paper will help shape personnel competencies in times of constant change.

The paper consists of an introduction and four sections. The first one is a review of the literature concerning fostering organisational culture and innovation at universities. The second section presents research methods, and the third one discusses the results. The work is completed with conclusions in the fourth section.

## Building a culture for innovation–a literature review

According to Khazanchi, Lewis, and Boyer [9], innovations that (paradoxically) require flexibility, improvements, and control are necessary to those in charge of organisations. The authors point out the crucial impact of organisational culture, which is the key to innovation management despite being complex and amorphous. Hence, to facilitate the immersion into the subject matter, a definition of organisational culture should be provided, which has been widely discussed in the literature.

Although Pettigrew [10] is generally believed to have introduced the notion of culture to the theory of organisation, it has been with social sciences–sociology and anthropology in particular–almost from the very beginning of these disciplines [11]. Matters related to organisational culture are interdisciplinary in nature [12]. Deshpande and Webster [2] described culture as a model, shared values, and beliefs that help understand how an organisation functions and thus provide them with standards of conduct. Barney [3] explained that not only does organisational culture reflect who the employees, customers, suppliers, and competitors are, but it also defines how the company will work with them. A strong culture helps employees understand the business strategy, motivates, and improves social relations among its members through mentoring. Therefore, if the success of a business strategy depends on conduct, the organisation needs the support of culture [4]. Moreover, culture determines what types of people are attracted by the organisation and who can be successful in it [13].

This approach is reflected in the definition by Hofstede [14], for whom organisational culture is ‘the collective programming of the mind’. Shared values and the strength of how much the values are shared by members of an organisation are at the core of an effort to build organisational culture. Internalisation of organisation’s values should lead to consistent goals of managers and individual employees [15]. This way, the framework of competing values of Quinn and Rohrbaugh [16] was developed. It is a concept according to which managers have to make choices that reflect two types of tensions within organisations: internal vs external and control vs flexibility. This two-dimensional representation yields four types of culture.

Adhocracy culture has a dynamic and creative work environment; employees take risks, and leaders introduce innovative solutions. Experiments and innovation facilitate growth. Lunch of new products and services is a success. Freedom and initiative of individuals are valued.

Clan culture is flexible and focused on internal maintenance, which determines relation-building behaviour. It is associated with the family and involves a friendly, almost family-like atmosphere. The leader is considered a mentor. The organisation is held together by loyalty and tradition. Success comes from the attention to customer’s and employees’ needs. Teamwork, participation, and consensus are the core values.

The third type of culture is market culture. Its characteristics are control and external maintenance, which results in highly competitive behaviour. Market culture drives actions towards results and completion of work and tasks. The pursuit of objectives integrates the organisation. Leaders take essential decisions themselves. Success is defined as increased market share and better financial results.

The last type is hierarchy culture focusing on control and internal maintenance. Behaviour should be predictable and ensure smooth operation. Hierarchy culture means formalised and structured work environment. Employees follow procedures to complete tasks. The core values are effective coordination and organisation. Success is defined as good planning and low costs.

Although all organisations exhibit some attributes of each type of culture, usually one of them dominates. Organisational culture can be diagnosed, and changes planned with the Organizational Culture Assessment Instrument by Cameron and Quinn [17]. The OCAI provides a diagnostic assessment of culture based on examining core values, shared assumptions, and common approaches to work. It is a classification approach to culture, and was designed to identify existing organisational culture as a prelude to cultural change [18]. Jaskyte and Dressler [19] covered the competing values framework and the impact of organisational culture, market orientation, and innovativeness on the financial results of competing businesses in their works.

The competing values framework was presented by Buschgens, Bausch, and Balkin [7] in an interesting way. They proposed value systems based on two dimensions, flexibility vs control and internal vs external orientation. The theory is intended to account for relationships between organisational culture and innovation. Culture itself describes the ideas behind organisational values. Managers can follow various strategies in line with the competing values scheme.

When discussing relationships between organisational culture and innovations, one should refer to the results of Naranjo-Valencia, Jimenez-Jimenez, and Sanz-Valle [20], who demonstrated that organisational culture is a clear indicator of the innovation strategy. They also noted that adhocracy culture fosters innovation, and hierarchy culture promotes imitation.

Therefore, one can conclude that organisational culture affects innovation and the success it generates to a great extent. Organisational culture is a vital leadership component and the key aspect of entrepreneurship [21].

Organisations strive for innovation. They invest in new products and optimise and streamline processes. The question remains, will they keep abreast of the growing pressure from their competitors? Most organisations operate in similar ways in their respective industries, and not many can achieve a permanent competitive advantage. Whatever the effort, the customer usually cannot see the features unique to individual organisations. Weis [22] and Christensen [23] believed organisations might collapse even with great products and good management. They argued that organisations fall because of the wrong decisions of their leaders.

The organisation’s effectiveness is affected by coherent operational culture and innovation [24]. Innovation capabilities are necessary for an organisation to draw on creative resources and new technologies. Still, innovation cannot be implemented always and everywhere. The source of innovation is creativity, but successful innovation needs more: specific social, economic, and political environment. Innovation requires proper organisational culture to thrive [25].

F. List appreciated the role of innovation in socioeconomic development back in the late 19^th^ century. The matter was revisited by J. A. Schumpeter, who contributed significantly to the theory of innovation and its role in economic development with waves of innovation. He believed key innovation occurs in cycles and drives growth [26]. Abundant literature offers many definitions of innovation based on classic propositions by Schumpeter [26], Porter [27], or Drucker [28]. The main cause of differences in the definitions is the focus on different aspects and components. Some definitions put the creation process first. Other ones emphasise the importance of implementation or the intensity of action. The definition proposed by Flynn and Chatman [29] seems to reconcile different approaches. It states that innovation is a combination of the creation of new ideas, their implementation, and an ongoing revision. This concept of innovation calls for a multifaceted search for innovation factors [30]. Stimulation of innovative activities requires a kind of organisational culture. Basic cultural attitudes that foster innovation are orientation towards the future, openness to changes, risk-taking, experimenting, creativity, trust, cooperation, mutual support, and tolerance of errors. Elements necessary to introduce innovation include a vision, ambitious challenges, and goals related to innovation, but also belief in action and atmosphere of deriving pleasure from work. Other ingredients are the autonomy to act, empowerment, freedom to come up with ideas, support for new ideas, and tolerance for discussion on new ideas [31]. C.A. O’Reilly believes that risk-taking, tolerance, teamwork, and speedy operation are the key to innovation culture [32, 33]. Innovation culture means general air of freedom both as regards specific behaviour (such as the right to make mistakes), and time and place of work or even appearance (informal clothing).

Innovation can be considered a tool for entrepreneurs to turn changes into an opportunity for the business or service [34]. Definitions of the concept of innovation offered by Zhao [34] Carmen, Fernandez-Alles, Martinez-Fierro [35], and Oyon [36] emphasise its three dimensions: innovation regarding a new product/service for a business unit, process innovation, and innovation as an inherent attribute of an organisation. Nevertheless, to make use of creative resources and consider innovation an organisational value (as was mentioned above), one needs motivation and leadership. Organisational culture is entwined with management. So to be able to change, an organisation needs to build adhocracy culture.

Therefore, innovation culture is a fundamental condition of organisational growth. It boosts entrepreneurship and is reflected in financial results [37, 38]. According to Hogan and Coote [39], innovation culture improves company performance as it promotes innovative behaviour, helps develop innovative products and services, and generates innovative solutions.

As opposed to some management theorists, Kirby and Ibrahim [40] believe that when directed towards entrepreneurship, organisational culture should not be understood as ‘more businesslike’ but has to promote innovation and creativity strongly. Hence, employees should be encouraged to take the initiative and be active, which improves sharing and cooperation, not only in business organisations but also in public administration institutions. Over the recent decade, researchers and managers identified organisational culture concepts in various environments to improve coherence and productivity at work [41]. In the academic context, culture can be defined as a set of specific values that leaders try to implement into organisations. Therefore, a better understanding of cultural matters found in groups and organisations is necessary to appreciate what happens there and determine potential priorities for leaders and leadership [42].

University organisational culture has been discussed broadly by Fralinger and Olson [8]. They defined it as the values and beliefs of university stakeholders (i.e. administrators, lecturers, students, management, and auxiliary personnel) based on traditions that can be transmitted verbally and non-verbally. The stakeholders can be divided into internal (current domestic and international students, graduates, and staff) and external (members of the relevant community, political jurisdictions, agencies, or press) [42]. Fralinger and Olson [8] pointed out the significant impact of values and beliefs on decision-making processes at universities and the formation of individual and organisational behaviour. Hence, university culture is reflected in the organisation’s personality. The architecture of the building, maintenance, student interactions and dress code all say a lot about university culture. It should be pointed out now that universities have characteristic features strongly correlated with their culture type.

As opposed to business organisations, university goals are often ambiguous and hard to measure. In this context, a university is a complex network, and the managers’ job is to make connections between its components [42]. Trust among the managers and staff is the key to efficient management. As universities follow the corporate model increasingly more, the understating of how corporate culture principles are compatible with university goals has become crucial. Researchers have investigated the issues of corporate leadership and promotion of the academic manager. Corporate governance is becoming the characteristic feature of higher education in the USA, while leadership increasingly shifts university culture towards corporate culture. The latter is oriented towards teamwork, employee support (including a broad skill development), fostering creativity, and constant change. The introduction of corporate principles to universities clearly shows that what used to be a part of a covert university curriculum, the subordinate role of learning to market demands, is redefining education policies at all levels today.

Note here that the university is one of the oldest institutions on earth. Despite its age, it has to retain its capabilities to innovate. What is more, some authors believe that universities need to innovate now more than ever to meet changing needs of the public [43]. University innovations are defined as more than inventiveness, transfer of knowledge, or commercialisation. Universities need to be set for evolution, modernisation, and changes in practices and offers [44]. Such effort entails developing an organisational culture that stimulates innovative and integrated changes through education, research, and public service [43]. Universities should not be merely places where people are trained but should shape behaviour and skills for innovation to grow with a direct influence on society [45]. The need for innovation at universities is indisputable. Challenges for higher education institutions include top-level training, creativity, and entrepreneurship. Innovative solutions are and will be in demand. Public goals of teaching and learning embedded in innovation must be prioritised. University innovativeness should be understood as creating own, separate products, services, and relevant processes that would offer high quality. Innovation in education, mainly higher education and research, require flexibility. When combined with entrepreneurial philosophy, learning, and new IT, they can restore the quality of life and social prosperity. Educational institutions (universities) should promote knowledge and skills for creating, innovating, problem-solving, emergency management, design thinking, critical thinking, teamwork, and prototyping for AI.

University innovations are associated with a change in the education system, innovative idea creation process promoted by education leaders, and university’s competitive edge. They can be discussed from the point of view of science (research) or the education process. Innovation in science means building an economy founded on knowledge and requires long-term investments and systemic support for businesses, universities, R&D centres, or the business environment. Stimulation of cooperation and implementation of solutions to resolve the issue of insufficient mechanisms for linking research and business ability to absorb new technologies, products, or innovative solutions is an essential part of the ecosystem. Education innovation is when some part of it changes (including management, work organisation, or teaching process innovations in the form of new teaching methods).

An innovative university creates a creativity-friendly culture. It is a place where interesting ideas are appreciated, and creative thinking is fuelled [46]. According to the OECD’s [47] innovation classification, an innovative university can implement product (educational offer), process (teaching process), marketing (social media use), organisational (structural changes, workplace organisation changes), and management (changed planning methods, management styles, or control) innovations.

Over the last 15 years, culture became a common way of perceiving and describing the inner world of any organisation. It is a method for differentiating the ‘personality’ of one organisation from another. Most researchers have come to accept the fact that organisational culture becomes more socialised, and ethics is its intrinsic component [48, 49]. Leadership, managers in particular, are of crucial importance when it comes to the shaping of innovation organisational culture both in business and at universities. Their leadership style should promote changes rather than maintain the structure and prevent conflicts between the organisation and creativity [50, 51]. It means that the actions taken in the organisation do not stimulate creativity. Additionally, leadership requires avoiding types of behaviour that are often at variance with traditional forms of management and organisational structure [52, 53].

The literature analysis systematised relevant knowledge and helped identify factors that determine the formation of innovation-friendly organisational culture. This way, drivers of innovation capacity could be represented as a model.

**Fig 1:**
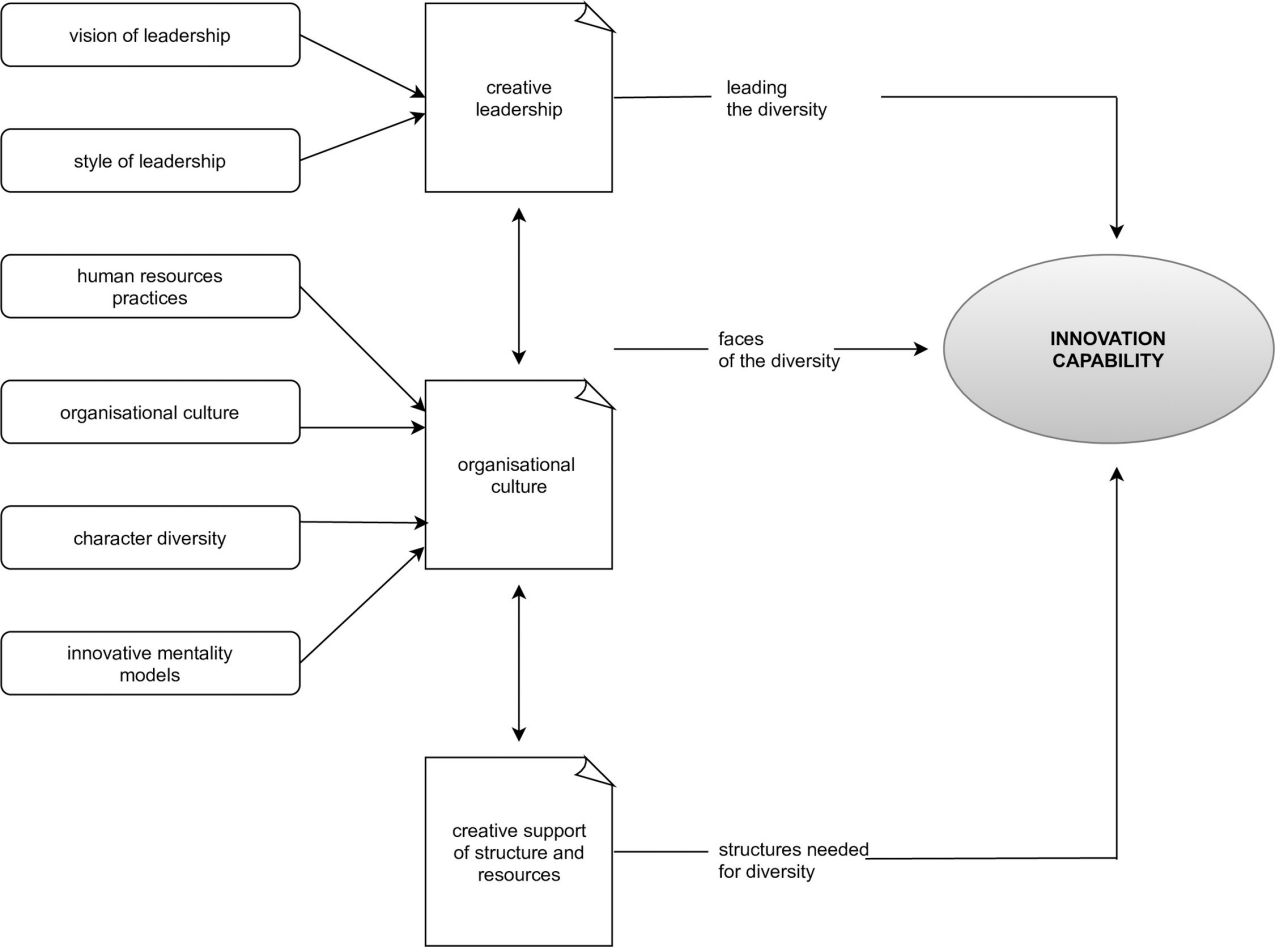
Components affecting the innovation capabilities of organisations (Original work based on Hynes, Mickahail [54]).

Fig 1 presents a model of components that shape the innovative capabilities of universities, business, public administration, or NGOs in light of the above.

The presented model of factors forming the innovation capabilities of universities indicates that managers (some of whom are often lecturers as well) should better pay heed to leadership, organisational culture, and support for structure and resources. With these areas diagnosed, one can design changes to create the innovative capability for the organisation.

## Methods

The public sector is one of the most interesting and, therefore, often addressed research areas. Various concepts and models have been proposed in research on the relationships between organisational culture and intellectual capital [6, 15, 55]. Additionally, Guthrie and Dumay [6] have demonstrated that the public is interested in the public sector, and the main difference between the public and private sector is the lack of normative research. Therefore, the present research focuses on analysing organisational cultures at selected universities in selected countries. Every country has its national culture that is part of the cultural universe. The cultural universe comprises pan-human symbolic systems, such as language, art and literature, knowledge and science, religion, and customs. The shaping of the cultural universe is conditioned by mechanisms emerging from the species-specific abilities of humans to create symbolic systems. The shaping and functioning of national cultures are related to separating some parts of the universe as a culture of a specific group. This way, its members can tell themselves from other groups with different cultures [56]. Therefore, the countries selected for the study were chosen due to their different cultures; two were from Western Europe and two from Eastern Europe.

**Fig 2:**
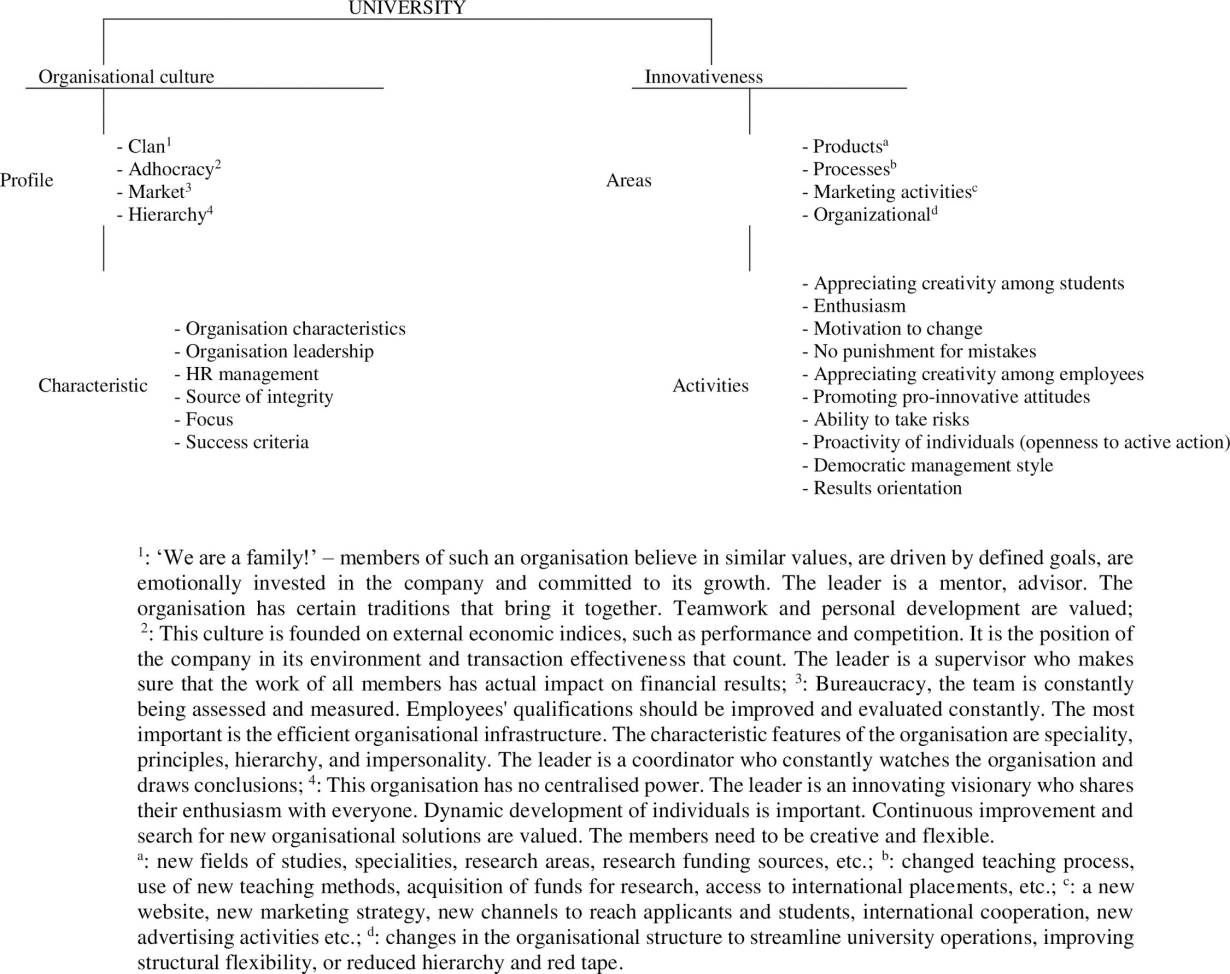
Main components of the original research.

To investigate the relationship between organisational culture and innovativeness of universities, we designed an original survey questionnaire [S1 File]. Its main components are shown in Fig 2.

To diagnose organisational culture, we used the Organizational Culture Assessment Instrument developed by K.S. Cameron and R.E. Quinn, which focuses on the basic characteristics of an organisation that reflect its culture profile. Each of the six questions in the questionnaire had four responses. Respondents distributed 100 points among these responses depending on the degree to which each response reflected the situation at their university. They should assign the highest score to the response most relevant to their university. Respondents assessed the current situation and the desired situation that they believed to be the most appropriate for the university.

The input from the first part of the work (literature review, Fig 1) was used to identify components that affect the formation of innovation-friendly organisational culture at universities. Respondents then used a five-point Likert scale to identify actions and assess whether they had the greatest or the minimal impact on the shape of organisational culture fostering innovation (1 for the least influential action and 5 for the most influential one). For the purposes of the paper, ‘action’ means effort made (activity engaged into) in areas identified by the bibliometric analysis as beneficial for university innovativeness. The evaluated actions were lack of punishment for mistakes, reliance on the familiar (old), promotion of pro-innovative attitudes, appreciation of employee creativity, appreciation of student creativity, proactivity of individuals, result-orientation, enthusiasm, risk-taking ability, motivation to change, and democratic leadership. The same scale was used to identify areas where new solutions were implemented over the recent four years in the universities. Values from 2 to 5 meant that such solutions were implemented to a small, medium, significant, and very significant degree, respectively, and value 1 meant no such solutions.

The questionnaire was delivered to representatives of universities in the selected countries (Ukraine, Austria, Germany, and Poland) who conducted the survey and returned completed questionnaires. The survey involved students because it is much easier for them to assess the educational mission and teaching process than research activities, which are difficult to evaluate. Moreover, an innovative university implements new solutions; its actions are aimed at creativity and entrepreneurship. These activities hinge on lecturers (educators) to a significant extent. Who can better evaluate this area than a student? Self-assessment is the most difficult method. Managers and staff often assess themselves as they would like to be, not as they are. The respondents received paper questionnaires. They were informed that the participation was voluntary. Our study did not include minors. The research did not require an ethics statement.

As it was a pilot study, the sample was purposive. It means we delivered the questionnaires to four partner universities that teach economics. The sample was selected to consist of respondents who were familiar with the subject of organisational culture. Hence, the questionnaires were delivered to students of management, who were second year second cycle degree programme students in the academic year 2019/20 (have studied for at least four years). The curriculum for the respondents included courses that involved analysis and diagnosis of organisational culture (i.e. organisation development, introduction to organisational culture, analysis and diagnosis of organisational culture, and management system design), and all of the participants had completed them. All the respondents participated voluntarily. The total number of returned questionnaires was from Germany 43, from Austria 34, from Ukraine 52, and from Poland 27, which was not less than 30% of the distributed questionnaires. We employed purposive sampling of students to facilitate designing grassroots changes to contribute to innovation-friendly organisational culture at universities.

**Fig 3:**
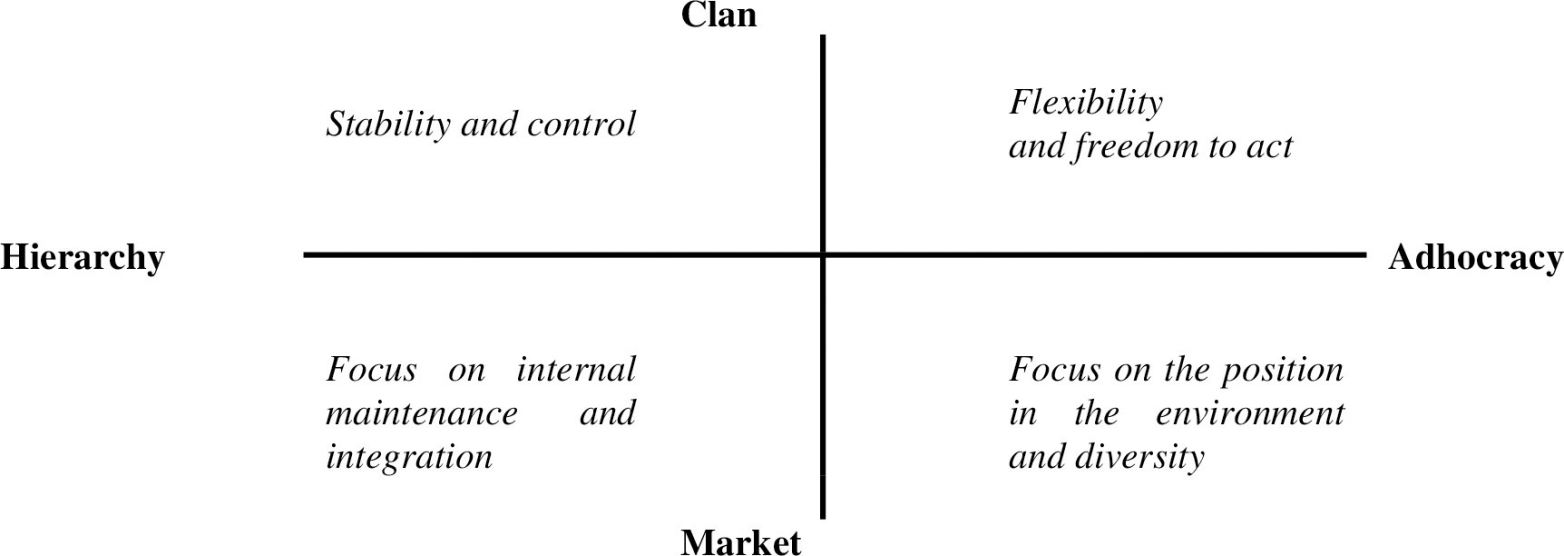
Cameron’s and Quinn’s organisational culture profile diagram.

The analysis was conducted in Dell Statistica v. 13.1 (StatSoft Polska). The results were described using basic statistics. First, we normalised data from the Likert [57] rating scale using Kaufman’s and Rousseeuw’s [58] formula. We then used Spearman’s correlation coefficient and Kendall’s W to calculate correlations for two and more order relations, respectively. Results were analysed with Cameron’s and Quinn’s [17] organisational culture profile diagram, as shown in Fig 3.

The following areas were used in the comparative analysis of cultures in the investigated universities: general characteristics of the organisation, leadership style, personnel management style, organisation integrity, key areas in the organisation (focus), success criteria.

## Organisational culture at selected universities–results

**Fig 4:**
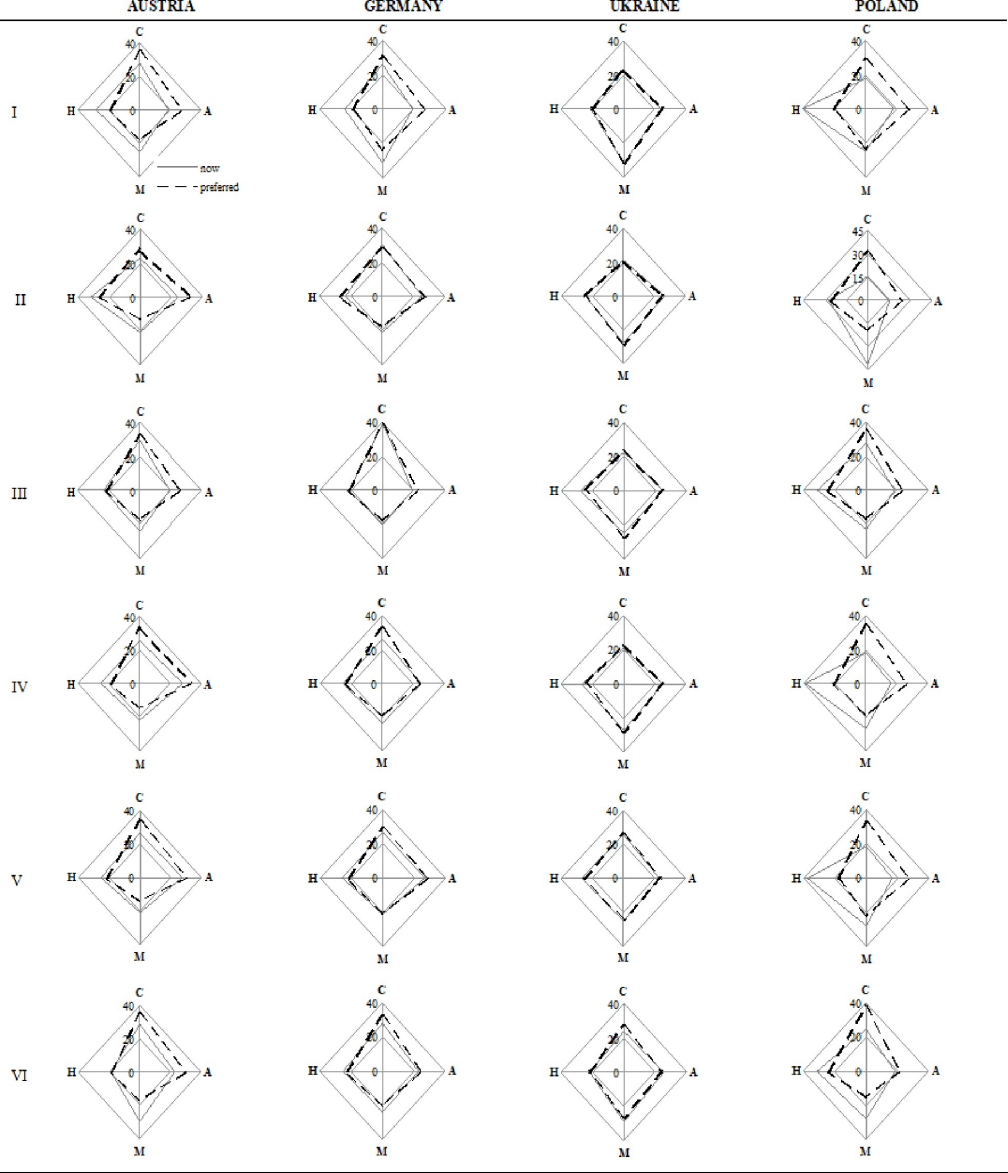
Mean culture profiles for each area in the selected countries. Areas: general characteristics of the organisation (I), leadership style (II), personnel management style (III), organisation integrity (IV), key areas in the organisation (V), success criteria (VI). Profile: Clan (C), Adhocracy (A), Market (M), Hierarchy (H).

The first stage of the research was a diagnosis of organisational culture types with Cameron’s and Quinn’s assessment instrument. Mean organisational culture profiles were determined for six areas of university organisations as presented in Fig 4.

An analysis of the summary on Fig 4. reveals that the Ukrainian university is dominated by market culture, and it is one of the countries where the existing and desired cultures overlap. The dominant culture at the German university is market as well, But the respondents indicated adhocracy and clan as the desired cultures. The university in Austria is dominated by hierarchy culture with clan and adhocracy as the desired cultures. The most hierarchised culture was found at the Polish university. The Fig 4. shows that the existing state is much different from the desired culture.

When analysing leadership, personnel management, and organisation integrity, one can notice that the existing and desired cultures are identical for the Ukrainian and German universities, which means that the organisational cultures are shaped correctly there. The most significant differences can be found in Poland. Austria has only minor differences. Polish universities focus on results, task completion, and pursuit of goals. The desired state is a creative work environment (adhocracy) and a friendly atmosphere where the leader is perceived as a mentor (clan). The integrity of the Ukrainian, Austrian, and German universities stems from loyalty, commitment to innovation, and hierarchy. The Polish universities is oriented towards formal principles and regulations intended to ensure efficient functioning. The respondents indicated experiments, innovative solutions, and teamwork as a means to achieve organisational integrity. They pointed at adhocracy and clan cultures (found in the other countries) as the desired ones for integrity and success criteria.

In Poland, the focus is on stability and invariability, which does not promote change, innovation, and growth. The policies in the other countries focus on competition-centred actions, personal growth, acquisition of new resources, and new challenges.

**Fig 5:**
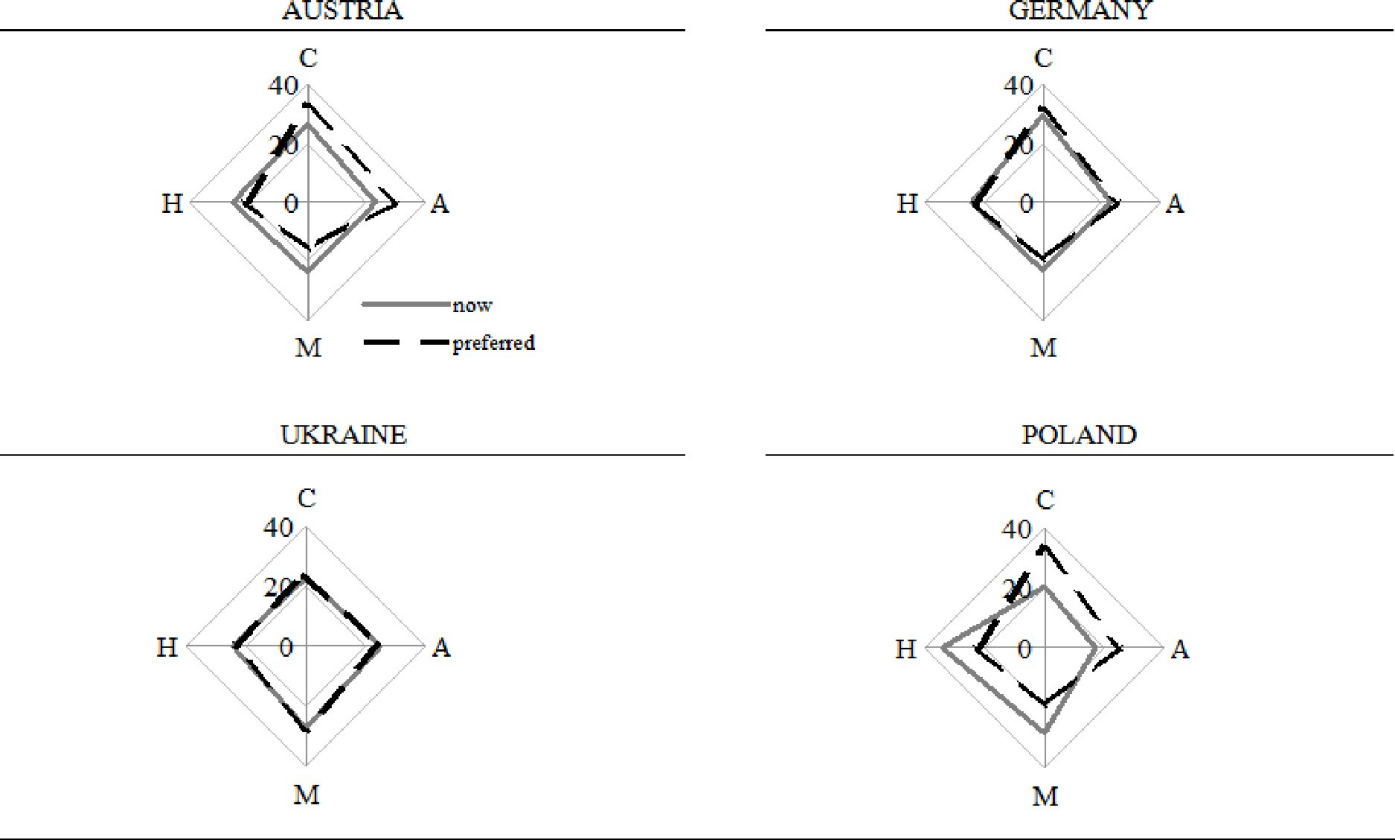
Mean organisational culture profiles in the selected countries. Areas: general characteristics of the organisation (I), leadership style (II), personnel management style (III), organisation integrity (IV), key areas in the organisation (V), success criteria (VI). Profile: Clan (C), Adhocracy (A), Market (M), Hierarchy (H).

To summarise this part, we propose conclusions regarding the mean organisational culture profiles in Fig 5. The German and Austrian universities exhibit characteristics of market, adhocracy, and clan cultures. Nevertheless, in both cases, there is a gap between the reality and desired state (greater at the Austrian university). Respondents from the German and Austrian universities defined the desirable state as clan and adhocracy cultures with a reduced hierarchy and market orientation. On the other hand, the Polish university has the most hierarchised culture of all the investigated universities. The respondents would like it to change.

**Fig 6:**
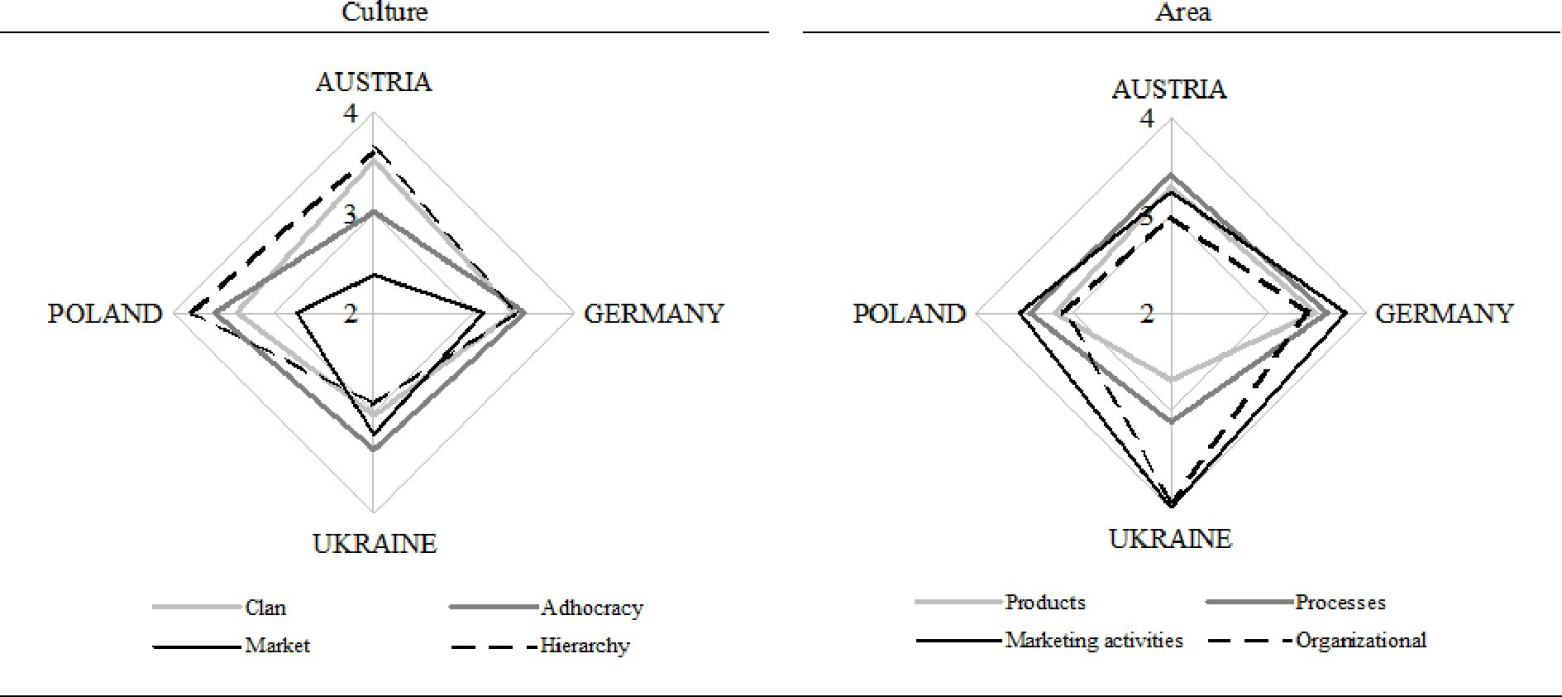
Type of culture favouring innovation and areas where innovation was introduced from 2015 to 2019 in the selected countries.

The next question in the survey questionnaire concerned the type of culture with the greatest impact on the university innovativeness. The respondents could choose from clan, market, hierarchy, and adhocracy cultures. At the next stage of the research, we checked in what areas the universities implemented innovation. The respondents could choose from among products (such as new fields of studies, specialities, research areas, or research funding sources), processes (such as changed teaching process, new teaching methods, acquisition of funds for research, or access to international placements), marketing (such as a new website, marketing strategy, channels to reach applicants and students, international cooperation, or new advertising activities), and organisational and structural solutions (changes in the organisational structure to streamline university operations, improving structural flexibility, or reduced hierarchy and red tape). The results are presented in Fig 6.

According to Fig 6, adhocracy and market cultures have the most significant impact on university innovativeness. The Ukrainian university is the leader in new organisational and marketing solutions. The German university is the most active in marketing innovation and then in processes and products. New organisational solutions are the least popular. Poland and Austria exhibit the smallest degree of innovativeness. Note that product, process, marketing, and organisational innovations were not considered introduced to a large extent in none of the countries.

The next question in the questionnaire concerned actions with the highest and the lowest impact on the establishment of innovation-friendly organisational culture. The ranking list of these activities is shown in Table 1.

**Table 1:** Ranking lists of measures to promote innovation-friendly organisational culture at universities.

Activities (abbreviation)	Ranking lists			
	AUSTRIA	GERMANY	UKRAINE	POLAND
Appreciating creativity among students (AS)	AS	AE	R	AE
Enthusiasm (E)	E	M	PA	AS
Motivation to change (M)	M	AS	AS	M
No punishment for mistakes (N)	N	PP	AE	E
Appreciating creativity among employees (AE)	AE	E	D	PP
Promoting pro-innovative attitudes (PP)	PP	PA	PP	PA
Ability to take risks (A)	A	A	E	A
Proactivity of individuals (openness to active action) (PA)	PA	D	A	R
Democratic management style (D)	D	R	M	N
Focus on results (R)	R	N	B	D
Reliance on what is familiar (old) (B)	B	B	N	B

Regarding actions with the greatest and least impact on the establishment of innovation-friendly organisational culture, respondents from all the universities indicated appreciation of students’ creativity among the three key actions. In Austria, they also indicated enthusiasm and motivation to change. Respondents from the Polish university also indicated motivation to change as an important action promoting innovation-friendly organisational culture. They believed the appreciation of employees’ creativity to be the paramount action. Respondents from the Ukrainian university believed the focus on results and proactivity of individuals to be the key drivers of innovation. An in-depth analysis of these results suggests that the respondents believed reliance on what is familiar, focus on results (all countries except Ukraine), and democratic leadership style to contribute the least to the establishment of innovation-friendly organisational culture. The remaining actions were considered as contributing to innovation-friendly culture.

Results of correlation significance tests for the ranking lists of actions driving innovation-friendly organisational culture on universities are summarised in Table 2.

**Table 2:** Results of Spearman’s rho correlation coefficient and Kendall’s W concordance coefficient significance tests (p-value) for the ranking lists of actions contributing to innovation-friendly organisational culture.

Specification	GERMANY	UKRAINE	POLAND
AUSTRIA	0.029	0.690	0.009
GERMANY		0.402	0.000
UKRAINE			0.359
Kendall’s W	0.008		

The verification of the significance of Kendall’s W concordance coefficients for all the ordered sets demonstrates that the respondents agreed on the order (importance) of actions contributing to the establishment of innovation-friendly organisational culture. In pair comparisons, the only statistically insignificant Spearman’s rho coefficients are the ones for the comparison of the ranking list for the Ukrainian university with any other ranking list (this means no monotonic relationship between the ranking lists). Hence, we conclude that the importance of actions contributing to the establishment of innovation-friendly organisational culture is the same at the universities in Austria, Germany, and Poland.

We then asked the respondents the following questions:

- Does organisational culture affect the innovativeness of universities?
- Does formalisation hinder the establishment of innovation-friendly culture?
- Does hierarchy hinder the establishment of innovation-friendly culture?

Results are shown in Table 3.

**Table 3:** Does organisational culture affect innovation at universities and do formalisation and hierarchy make it difficult?

Responses		AUSTRIA	GERMANY	UKRAINE	POLAND
Does organisational culture affect the innovativeness of universities?					
	No	2.94	1.92	23.08	0.00
	yes	88.24	78.85	75.00	76.47
	I have no opinion	8.82	1.92	1.92	2.94
Does formalisation hinder the establishment of innovation-friendly culture?					
	no	20.59	17.31	53.85	3.70
	yes	41.18	44.23	40.38	88.89
	I have no opinion	38.24	21.15	5.77	7.41
Does hierarchy hinder the establishment of innovation-friendly culture?					
	no	17.65	23.08	55.77	11.11
	yes	50.00	44.23	38.46	70.37
	I have no opinion	32.35	15.38	5.77	18.52

A significant share of the respondents (not less than 75%) in each university indicated that organisational culture impacts university innovativeness. The vast majority of the respondents at the Polish university believed formalisation and hierarchy to hinder the establishment of innovation-friendly culture. Other respondents disagreed. A little over half of the respondents from Ukraine judged these factors as not affecting the establishment of innovation-friendly culture.

Chi-square independence tests indicated that respondents’ opinions on whether or not organisational culture affected university innovativeness and formalisation or whether hierarchy hindered the establishment of innovation-oriented culture depended on the university from which the respondents were. Cramér’s Vs are 0.229; 0.343, and 0.274, which indicates moderate relationships.

## Discussion

With the present research, we identified organisational cultures at universities in Austria, Poland, Germany, and Ukraine. Organisational culture seems to be an important factor for innovation. Results of the research show that innovation is bolstered by such aspects of organisational culture as flexibility and focus on external maintenance and market and clan culture models. These types of culture were found to prevail in the Ukrainian and German universities. Polish and Austrian ones were dominated by hierarchy culture. It hinders innovative processes in organisations. The respondents listed the appreciation of student and employee creativity, enthusiasm, motivation to change, tolerance for mistakes, promotion of innovation-friendly actions, risk-taking ability, and proactive attitudes of individuals as actions taken to establish open innovation culture. The fundamental aspect of innovation culture is the openness to new ideas and technologies that can come from within or without the organisation. It is particularly important for the initiation of an innovation process. As innovative activity usually entails a tremendous risk of failure, innovation culture also encompasses the atmosphere that encourages risk-taking and inclination towards risk. The degree of innovation culture determines the extent of risk taken related to experiments, and resulting in creative errors and learning. None of the investigated universities was found to be dominated by adhocracy culture. One could conclude that they are not very innovative (only the Ukrainian university exhibited a mixed culture identical to the desired culture, but the research did not look into the causes of this. The innovativeness of Ukrainian universities was not identified).

Note also that the investigated universities do not implement technology and non-technology innovation to a large extent, which inhibits the growth of innovation at the universities.

**Fig 7:**
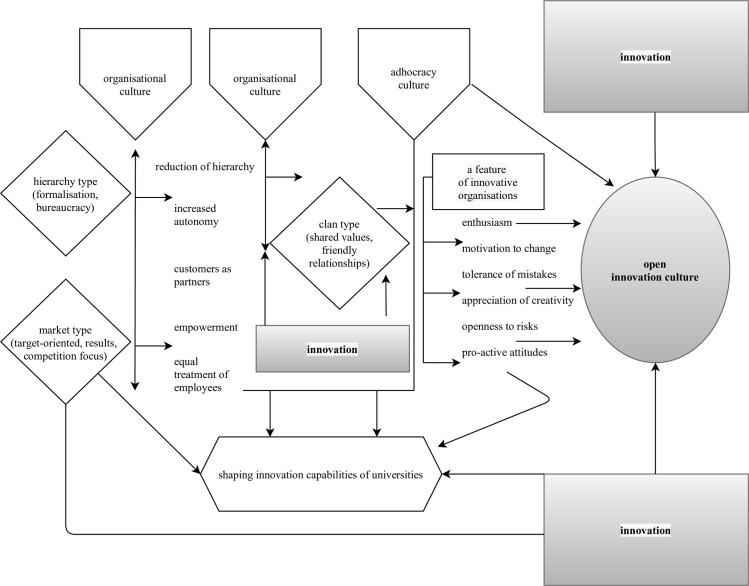
A model of how innovation-friendly organisational culture is formed at universities.

These deliberations facilitated an attempt to build a model of how organisational culture (of open innovation) is shaped that determines the innovativeness at the universities, as shown in Fig 7.

According to the research, the Polish and Austrian universities were dominated by hierarchy and market culture, while the German and Ukrainian universities had aspects of clan and adhocracy culture. Therefore, we recommend diagnosing the organisational culture of the specific university before any changes are planned. The literature review suggests that adhocracy culture (aided by the implementation of innovation, motivation to change, tolerance for mistakes, appreciation of creativity, and support for proactive attitudes) will contribute to the establishment of organisational culture that fosters university innovativeness. All the respondents agreed that organisational culture affects university innovation. They further indicated that openness and support from leaders who are mentors were lacking, and focus on targets should be reduced. When a university organisational culture diagnosis indicates hierarchy or market culture, small steps should be taken towards clan culture and adhocracy. The actions should be aimed at increasing autonomy, reducing hierarchy, and treating customers as partners. Clan culture supplemented with innovation will be the cornerstone for further changes to establish adhocracy culture.

The very nature of innovation changed over recent years. The complexity of products, availability of inexpensive skilled workers in developing countries, and growth of special talents in various parts of the world specific to their respective regions [59] encouraged organisations to apply out-of-the-box solutions to acquire innovation resources. Innovation-friendly organisational culture involves the use of the best ideas and people wherever they may be. It is true for both new products and services and new processes, business models, or methods and practices [60]. Changes in organisational culture should be designed using new ideas from both within and without the organisation. One should strive to achieve a structure that accepts ideas originating from within the company and those that come from outside, from resources other than the current operations [61]. The first Schumpeter’s model in which a single business puts innovation on the market was replaced with a model based on the cooperation of many parties to transform a new idea into a product or service. The proposed innovation model presents an interactive process where users are not on their own but work together as teams based on trust, meeting in application-focused partnerships, and participating in intensive, interactive networks [62].

## Conclusions

The paper achieved the research goal to diagnose organisational culture at one university from each Austria, Poland, Germany, and Ukraine. We further analysed the impact of organisational culture on the innovativeness of the universities.

The research concluded that the Polish and Austrian universities are each dominated by hierarchy and market cultures. The German university and the Ukrainian University exhibit all culture types, with clan and adhocracy cultures dominating. The analyses further demonstrated that:

- although it was the least apparent in the investigated institutions, adhocracy culture has the most impact on university innovativeness;
- product, process, marketing, and organisational innovations are not implemented to a large extent by the investigated universities, which harms university innovativeness;
- the Ukrainian university is the leader in new organisational and marketing solutions. The German university implements new solutions in marketing innovation and then in processes and products the most. New organisational solutions are the least evident. The university in Poland and the one in Austria are the least innovative of all the investigated schools;
- one could venture that there is a relationship between hierarchical cultures at the Polish and Austrian universities and a shortage of new solutions, which would confirm the thesis that hierarchy culture hinders university innovativeness;
- at the Polish university, formalisation and hierarchy slow down the establishment of innovation-friendly culture, while as regards the other countries, only 50% of the respondents from Ukraine believed that culture is not crippled this way;
- innovation-friendly university culture can be established at universities by change planning, openness to new ideas and creativity (both among students and staff), enthusiasm, risk-taking ability, team building, and implementation of technology, marketing, and organisational innovation.

The conclusions were used to construct a model for forming innovation-friendly organisational culture at universities. The model responds to the research question. It contains guidelines on how to shape organisational culture to foster innovativeness at universities. The research identified relationships between organisational culture and university innovativeness. It also indicated components that form the innovation capabilities at universities as its contribution to the theory of management.

In practice, the guidelines can help shape university organisational culture bottom-up. The model can be a lodestar for internal and external stakeholders.

The survey was intentionally addressed to students so that the conclusions can be applied to the functioning of academic teachers and administration. The limitation of the research is that it involved only four selected universities, so that no general conclusions can be drawn. Nevertheless, the results can be a foundation for future research on the relationships between organisational culture and university innovativeness as perceived by students and staff. Future research can investigate university innovativeness from the point of view of students and teachers. Moreover, research on barriers for promoting adhocracy culture at universities would be interesting. Further in-depth research on why universities do not use organisational culture to create innovativeness should also be considered. Finally, research regarding the cultural environment of specific countries and their impact on university innovation could be just as interesting. Moreover, future research can attempt to tackle the research question of whether the impact of organisational culture on innovations is affected by the type of organisation.

## Supporting information

S1 File Survey questionnaire. (DOCX)
