# Supplementary material for: Finding links between organisation’s culture and innovation. The impact of organisational culture on university innovativeness
Source: PLoS One. 2021 Oct 8;16(10):e0257962. doi: 10.1371/journal.pone.0257962 (PMC8500439; doi:10.1371/journal.pone.0257962)
Supplement: S1 File — (DOCX) [file pone.0257962.s001.docx]

**Part B**

1. **Does organisational culture affect the university's innovativeness?**
2. Yes
3. No
4. No opinion

| **Culture** | **1**  has no impact | **2**  has a limited impact | **3**  has a moderate impact | **4**  has a large impact | **5**  has a very large impact |
| --- | --- | --- | --- | --- | --- |
| Clan^[[1]](#footnote-1)^ |  |  |  |  |  |
| Market^[[2]](#footnote-2)^ |  |  |  |  |  |
| Hierarchy^[[3]](#footnote-3)^ |  |  |  |  |  |
| Adhocracy^[[4]](#footnote-4)^ |  |  |  |  |  |

1. **Assess which type of culture helps introduce innovation to universities (scale 1–5)**
2. **Which areas have had new solutions (innovations) introduced from 2015–2019 (scale 1–5)**

| **Area** | **1**  not  introduced | **2**  to a small degree | **3**  to a moderate degree | **4**  to a great degree | **5**  to a very great degree |
| --- | --- | --- | --- | --- | --- |
| Products^^[[5]](#footnote-5)^^ |  |  |  |  |  |
| Processes^^[[6]](#footnote-6)^^ |  |  |  |  |  |
| Marketing^^[[7]](#footnote-7)^^ |  |  |  |  |  |
| Organisational (structural solutions)^^[[8]](#footnote-8)^^ |  |  |  |  |  |

1. **Assess which of the following actions had the greatest and the smallest impact on the formation of innovation-friendly organisational culture (scale 1–5)**

| **Action** | **1**  has no impact | **2**  has a limited impact | **3**  has a moderate impact | **4**  has a large impact | **5**  has a very large impact |
| --- | --- | --- | --- | --- | --- |
| No punishment for mistakes |  |  |  |  |  |
| Reliance on the familiar (old) |  |  |  |  |  |
| Promotion of pro-innovative attitudes |  |  |  |  |  |
| Appreciation of employee creativity |  |  |  |  |  |
| Appreciation of student creativity |  |  |  |  |  |
| Proactive attitudes of individuals |  |  |  |  |  |
| Results orientation |  |  |  |  |  |
| Enthusiasm |  |  |  |  |  |
| Risk-taking ability |  |  |  |  |  |
| Motivation to change |  |  |  |  |  |
| Democratic leadership |  |  |  |  |  |

1. **Does the university diagnose its organisational culture?**
2. No
3. No opinion
4. Yes
5. **Does formalisation hinder the establishment of innovation-friendly culture at universities?**
6. Yes
7. No
8. No opinion
9. **Does hierarchy hinder the establishment of innovation-friendly culture at universities?**
10. Yes
11. No
12. No opinion

1. ‘We are a family!’ – members of such an organisation believe in similar values, are driven by defined goals, are emotionally invested in the company and committed to its growth. The leader is a mentor, advisor. The organisation has certain traditions that bring it together. Team work and personal development are valued. [↑](#footnote-ref-1)
2. This culture is founded on external economic indices, such as performance and competition. It is the position of the company in its environment and transaction effectiveness that count. The leader is a supervisor who makes sure that work of all members has actual impact on financial results [↑](#footnote-ref-2)
3. Bureaucracy, the team is constantly being assessed and measured. Employees' qualifications should be improved and evaluated constantly. The most important is the efficient organisational infrastructure. The characteristic features of the organisation are speciality, principles, hierarchy, and impersonality. The leader is a coordinator who constantly watches the organisation and draws conclusions. [↑](#footnote-ref-3)
4. This organisation has no centralised power. The leader is an innovating visionary who shares their enthusiasm with everyone. Dynamic development of individuals is important. Continuous improvement and search for new organisational solutions are valued. The members need to be creative and flexible. [↑](#footnote-ref-4)
5. Such as new fields of studies, specialities, research areas, or research funding sources [↑](#footnote-ref-5)
6. Such as changed teaching process, new teaching methods, acquisition of funds for research, or access to international placements [↑](#footnote-ref-6)
7. Such as a new website, marketing strategy, channels to reach applicants and students, international cooperation, or new advertising activities [↑](#footnote-ref-7)
8. Changes in the organisational structure to streamline university operations, improving structural flexibility, or reducing hierarchy and red tape. [↑](#footnote-ref-8)
